# Flagging urgent suspicion of lung cancer with AI CXR algorithms: optimising feature selection and performance

**DOI:** 10.1186/s13244-026-02438-7

**Published:** 2026-10-31

**Authors:** Sean F. Duncan, Katie Dudman, Shalom Odigbo, Jesus Perdomo Lampignano, Shamie Kumar, Neil Hawkins, Alex McConnachie, John D. Maclay, David B. Stobo, Mark Hall, David J. Lowe

**Affiliations:** 1 HealthTech Innovation & Translation Lab, University of Glasgow, Imaging Centre of Excellence, Langlands Drive, Glasgow, G51 4LB UK; 2 The Beatson West of Scotland Cancer Centre, 1053 Great Western Road, Glasgow, G12 0YN UK; 3 Robertson Centre for Biostatistics, School of Health and Wellbeing, University of Glasgow, Clarice Pears Building, Level 5, 90 Byres Road, Glasgow, G12 8TB UK; 4 West of Scotland Innovation Hub, NHS Greater Glasgow and Clyde, Glasgow, UK; 5 Radiology Department, Queen Elizabeth University Hospital, NHS Greater Glasgow and Clyde, 1345 Govan Road, Glasgow, G51 4TF UK; 6 Qure.AI, Mumbai, India; 7 Health Economics & Health Technology Assessment, School of Health and Wellbeing, University of Glasgow, Glasgow, UK; 8 Department of Respiratory Medicine, Glasgow Royal Infirmary, 84 Castle Street, Glasgow, G4 0SF UK; 9 Emergency Department, Queen Elizabeth University Hospital, Glasgow, NHS Greater Glasgow and Clyde, 1345 Govan Road, Glasgow, G51 4TF UK

**Keywords:** Lung neoplasms, Radiography (Thoracic), Artificial intelligence, Early detection of cancer, Radiographic image interpretation (Computer-assisted)

## Abstract

**Objectives:**

To investigate the impact of feature selection and thresholding on the performance of a commercial chest X-ray (CXR) algorithm for flagging urgent suspicion of lung cancer (USC) in a West of Scotland population.

**Materials and methods:**

The datasets comprised 2887 prospectively collected CXRs from general practitioners and outpatient routine care in the West of Scotland. An additional validation dataset included 983 retrospectively collected CXRs. The index test was an algorithm probability score; the reference standard was USC, derived from a blinded expert radiologist panel review of CXR. Three feature selection approaches were compared: radiologist-selected features with commercial thresholding of individual features, radiologist-selected features with regression weighting for the combined score, and a model using Akaike Information Criterion (AIC) and LASSO regression approaches for feature selection. Performance was evaluated at two thresholds: the Youden index and 15% positive flag rate.

**Results:**

Regression-based feature selection using the AIC and LASSO approaches resulted in a four-feature model (consolidation, nodule, tuberculosis and cavity) compared with the five features (cavity, nodule, mass, hilar enlargement and mediastinal widening) selected by clinical experts. Sensitivity was highest in the AIC/LASSO model, but was significantly impacted by a 15% flag rate threshold. Regression weighting for the combined score demonstrated superior performance compared to individual feature thresholds.

**Conclusion:**

In this study, regression-based feature refinement against an expert-derived reference standard outperformed clinically intuitive selection. Commercial CXR algorithms require local calibration and task-specific optimisation for USC detection. Workflow and post-market deployment considerations can be addressed by constraining positive flag rates through a unified model threshold.

**Key Points:**

***Question*** How should users select and combine AI chest X-ray algorithm features to achieve optimal deployment performance for detecting urgent suspicion of cancer in routine practice?

***Findings*** Regression-based feature selection outperformed expert-selected features, and combining features under a single regression-weighted threshold outperformed individual feature thresholds for urgent cancer suspicion detection.

***Critical relevance statement*** Optimising chest X-ray AI for urgent cancer suspicion requires data-driven, regression-based feature selection rather than clinical intuition; health systems therefore need local capability to validate and calibrate algorithms for their population and task.

**Graphical Abstract:**

**Figure:**
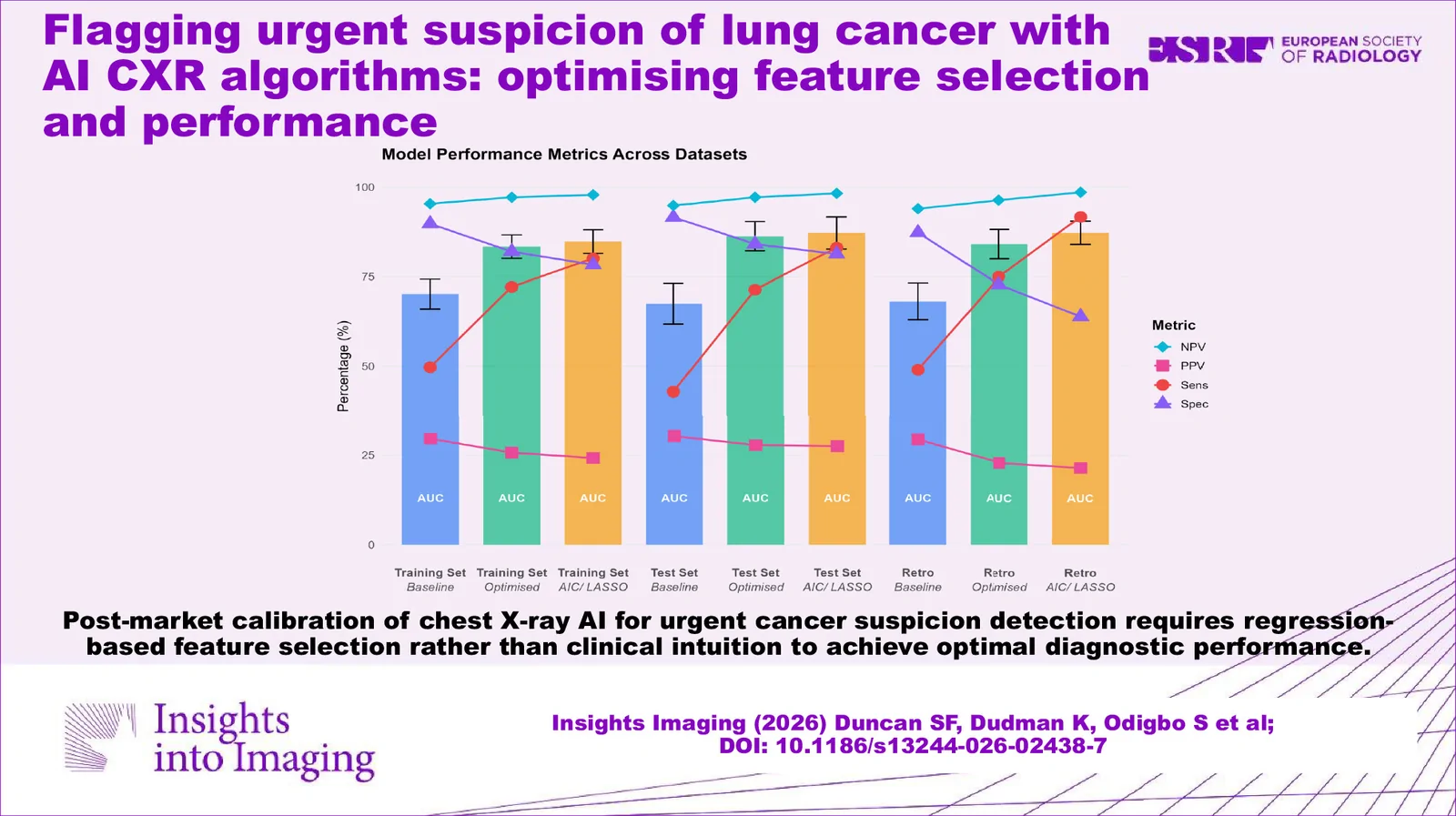

## Introduction

Lung cancer is a poor-prognosis cancer that disproportionately affects patients with underlying lung disease [1]. Early symptoms of lung cancer are often non-specific and challenging to discern from other lung diseases. Chest X-ray (CXR) is a readily available, cheap and non-invasive imaging technique that is often used as a first-line investigation for cardio-respiratory symptoms. In the UK, CXRs are the most frequently requested examination and are generally reported by Radiologists [2]. Reporting capacity is often outstripped by demand, commonly leading to a backlog of images and delays in the reporting of malignant findings and subsequent diagnosis of lung cancer [3, 4]. CXR artificial intelligence (AI) algorithms have been proposed as a potential solution to complement radiology workflows by triaging suspicious images for prioritised reporting [5]. These algorithms are trained on large imaging datasets that have been labelled with a range of pathologies. The output of these algorithms typically takes the form of feature-level probability scores and an image overlay with bounding boxes around abnormal areas to complement radiological interpretation.

Commercial vendors seeking to translate this technology into real-world clinical workflows have explored potential use cases in lung cancer pathways with mixed results [6–12]. Within lung cancer pathways, the CXR is fundamentally used to highlight cases of ‘Urgent Suspicion of Cancer’ (USC) and trigger further investigation through cross-sectional imaging modalities such as CT [13]. Therefore, an algorithm that occupies a complementary function within this pathway should be evaluated for its performance in detecting USC. Many commercial CXR algorithms do not provide USC as an output feature, instead focusing on clinically derived stem features such as ‘mass’, ‘cavity’ or ‘opacity’. However, the relationship between these features and the clinically relevant triage-construct of the radiologist determined USC must be investigated to understand post-market feature selection and thresholding for optimal local performance.

In this study, we aim to identify optimal AI CXR feature and threshold configurations for flagging USC in retrospective and prospective datasets using an expert consensus reference standard.

## Materials and methods

RADICAL was granted REC approval in August 2023 from the North West—Greater Manchester West Research Ethics Committee (REC 23/NW/0211), has been registered on clinicaltrials.gov (NCT06044454), and the protocol has been published [13].

**Fig. 1:**
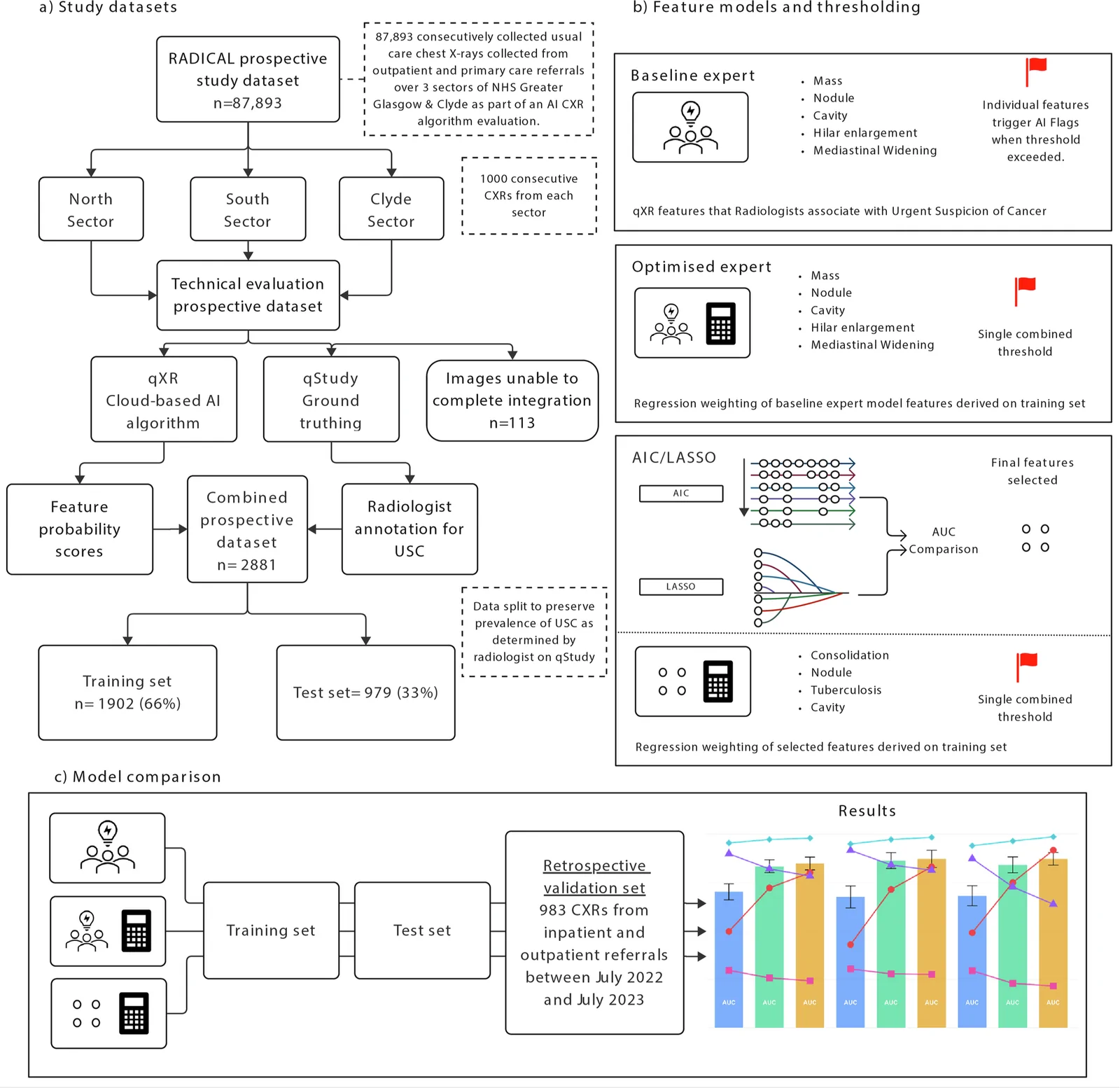
Study schematic. **a** Creation of study datasets; **b** Modelling relationships between features and USC; **c** Model comparison

This technical evaluation and the included prospective and retrospective datasets were a planned component of the RADICAL (Radiograph Accelerated Detection and Identification of Cancer in the Lung) study [14]. Figure 1 shows the overall study structure. This study was reported in accordance with the STARD AI (Standards for Reporting of Diagnostic Accuracy Studies-Artificial Intelligence) guidelines for diagnostic accuracy studies involving AI [15].

### Datasets

For the prospective dataset, the first 1000 consecutive CXRs were collected from outpatient and primary care referrals in each of the three organisational sectors of NHS Greater Glasgow and Clyde (NHSGGC), undergoing AI implementation between October 2023 and October 2024. Only patients aged ≥ 18 years were eligible; inpatient CXRs were excluded.

For the retrospective external validation dataset, 1000 CXRs were randomly sampled from patients aged ≥18 years who underwent outpatient and inpatient imaging (excluding primary care) in NHSGGC between July 2022 and July 2023. Cases were proportionally stratified by age group (< 60 vs. ≥ 60 years), sex, and month of acquisition prior to randomisation to ensure representative distribution in an all-cause healthcare population. Inpatient imaging was included to assess the generalisability of model performance to all hospital contexts. The sample sizes for both datasets were pre-specified in the RADICAL study protocol. For modelling, the prospective dataset underwent a 66:33 split into training and test sets, using stratified sampling to maintain the proportion of USC in each set.

### qXR software and features

qXR (version 4.0, 2023) is a commercial CXR algorithm developed by qure.ai. It is a CE class IIB medical device that generates probability scores for 22 different abnormalities (S1). qXR image interpretation generates a probability score for each feature; these scores were used for the feature selection and thresholding work below.

### Reference standards

For all datasets, the reference standard was established using the vendor-provided platform ‘*qStudy’* (S2). Each CXR was de-identified and underwent independent double-reading by a pool of five post-fellowship radiologists (5–10 years’ experience) with DICOM standards in line with RCR recommendations [16]. Disagreements were adjudicated by 1 of 2 senior radiologists (7 and 20 years’ experience). Readers were blinded to clinical history, prior imaging, AI outputs, other annotator opinions and disease prevalence. Annotators recorded abnormal features and provided a binary judgement on the presence of USC.

### Modelling features for urgent suspicion of cancer

#### USC feature selection

Three feature selection methods were compared:

##### Baseline expert model

Radiologists’ (M.H. and D.S.) in consensus with qure.ai, based on feature labels considered clinically relevant for USC detection. A positive AI flag was generated when any feature score exceeded its corresponding threshold.

##### Optimised expert model

Baseline expert features but applied logistic regression to derive weighted contributions and a single combined threshold.

##### Akaike Information Criterion (AIC)/LASSO model

For this model, we selected 17/22 qXR features and used two well-established feature selection algorithms, AIC, and LASSO regression, to select the optimal combination of features for predicting USC in the training set. Five features were excluded from the feature selection algorithms on clinical grounds to reduce the risk of over-fitting (S4). For LASSO, 10-fold cross-validation derived optimal penalty terms: lambda.min (lowest deviance) and lambda.1se (highest lambda within 1 standard error of lambda.min).

AIC/LASSO candidate models were then compared for test-set area under the curve (AUC) using DeLong’s test with a Bonferroni correction. Where models did not differ significantly in AUC, the most parsimonious model (fewer features) was selected to reduce the risk of overfitting and minimise the calibration burden for local deployment. Logistic regression was then performed on the AIC/ LASSO features to derive weighted contributions and a single combined threshold.

### Performance statistics and threshold selection

Model performance will be reported through the area under the receiver operating curve (AUC), sensitivity, specificity, positive predictive value, negative predictive value and accuracy. For the baseline expert model, individual feature thresholds were defined by the vendor following an internal USC-targeted calibration exercise (S3). For other models, two thresholds were evaluated: (1) Youden index (optimal combined sensitivity/ specificity), and (2) 15% positive flag rate derived on the training set and applied to the test and retrospective datasets. The positive flag rate is defined as the proportion of all images that each model flags as USC. The 15% cut-off was chosen based on the local pathway radiology resource and will therefore vary in accordance with institutional capacity.

### Data analysis

Statistical analysis was performed by K.D., A.M. and S.D. on R version 4.4.0 [17] using packages ggplot2 [18] for figures, pROC [19] for ROC curves, glmnet [20] for regression and tidyverse [21] for data pre-processing and summarisation.

## Results

Table 1 demonstrates broad demographic details of the retrospective and prospective datasets, with major differences being demonstrated in referral source. In the prospective dataset, 2881 images were transferred to qXR and qStudy (919 in the North sector, 996 in the South sector and 966 in the Clyde sector), with 113 images not completing transfer to qXR for analysis (54 outside of the inclusion criteria (S1), 59 unable to fetch from the server). 17 of the 1000 retrospective cases were duplicates, leaving a total of 983. qStudy interobserver disagreement rates are reported in S5.

**Table 1:** Comparison of retrospective and prospective datasets.

		Prospective dataset		Retrospective dataset
		Training	Test	Validation
Number of images		1902	979	983
Median age (IQR)		61 (48–71)	61 (49–72)	64 (51–76)
Sex	Female (%)	1006 (52.9)	519 (53.0)	521 (53.0)
	Male (%)	896 (47.1)	460 (47.0)	462 (47.0)
Referral Source	Inpatient	0	0	632
	Outpatient	399	209	351
	GP	1503	770	0
USC by radiologist prevalence		7.9%	7.9%	9.8%

### Baseline expert model

The Radiologist selected qXR features for USC detection were Mass, Nodule, Cavity, Hilar enlargement and Mediastinal widening. AUC was similar across the training, test and retrospective datasets (70.2, 67.5, and 68.1, respectively). Sensitivity was less than 50% across all datasets with low per-feature sensitivity (S6).

### Optimised expert model

**Fig. 2:**
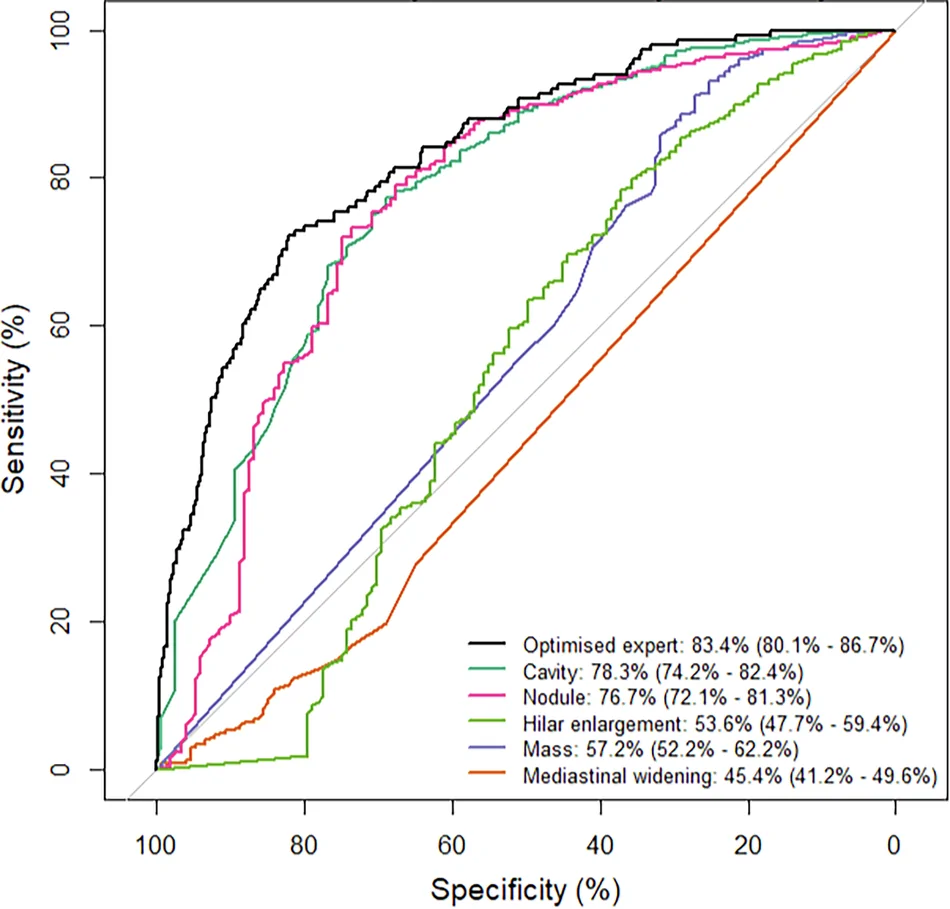
ROC curve for each individual feature in the baseline expert model alongside the optimised expert model

Table 2 demonstrates the output of the logistic regression across the five baseline expert model features. Cavity was the strongest feature in this model, with a 17-fold increase in the odds of USC when the threshold was breached. Simplified weighting was calculated by dividing each feature coefficient by the sum of all feature coefficients. Figure 2 demonstrates the ROC curve for each of the individual features alongside the ROC curve for the optimised expert model.

**Table 2:** Optimised expert model co-efficients, odds ratios and weightings of the optimised model. Mediastinal widening excluded as negative value

Feature (optimised expert)	Co-efficient	Odds ratio (95% confidence interval)	Simplified weighting
Cavity	2.9	17.4 (7.1–43.3)	35%
Nodule	2.4	11.2 (6.2–20.1)	29%
Mass	1.9	6.5 (1.7–25.8)	23%
Hilar enlargement	0.99	2.7 (1.2–6.0)	12%
Mediastinal widening	−0.05	1.0 (0.0–8.5)	n/a
Model intercept	−3.9		

### AIC/LASSO model

AIC stepwise feature selection and LASSO regression were performed on the 17 selected features: cavity, mass, nodule, fibrosis, elevated hemidiaphragm, hyperinflation, pleural effusion, atelectasis, rib fracture, tuberculosis, calcification, consolidation, mediastinal mass, mediastinal widening, hilar enlargement, blunted costophrenic angles and cardiomegaly.

The model favoured by the AIC was a 5-feature model containing: cavity, nodule, TB, consolidation and hilar enlargement. For the LASSO feature selection, lambda(min)resulted in 6 features: cavity, mass, nodule, TB, consolidation and hilar enlargement. Lambda(1se) resulted in 4 features: cavity, nodule, TB and consolidation. There was no statistically significant difference in AUC performance between all LASSO/AIC models (S7), and therefore, the lowest feature model (lambda(1se)) was selected in accordance with our pre-specified parsimony rule.

Table 3 demonstrates the output of a logistic regression model using the four selected features in the AIC/ LASSO model. Consolidation was the strongest performing feature in this model, with a 7.5-fold increase in the odds of USC when the threshold was breached.

**Table 3:** Data-driven model coefficients, odds ratios and weightings of the AIC/ LASSO model

Feature (AIC/ LASSO)	Co-efficient	Odds ratio (95% confidence interval)	Simplified weighting
Consolidation	2.0	7.5 (3.0–18.7)	31%
Nodule	1.8	6.3 (3.1–12.6)	28%
Tuberculosis	1.6	4.8 (1.7–13.1)	25%
Cavity	1.0	2.8 (0.9–8.6)	16%
Model intercept	−3.9		

### Model comparison: AUC

**Fig. 3:**
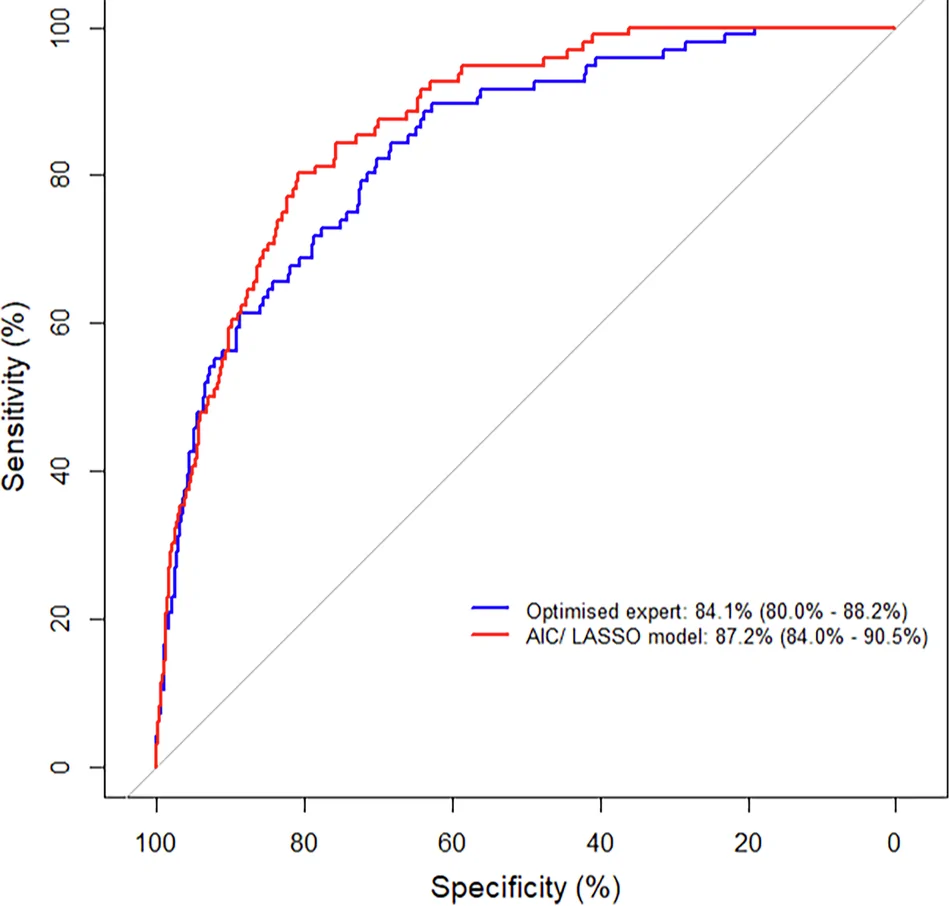
ROC curve comparison between the optimised expert and AIC/ LASSO model on the retrospective data

Table 4 demonstrates performance statistics across models, datasets and thresholds. Both the optimised expert and AIC/ LASSO models outperformed the baseline expert model for AUC across all datasets. There was no statistically significant difference between the optimised expert model and the AIC/ LASSO model for AUC across the training and test data using DeLong’s test (83.4% vs. 84.8 (*p* = 0.16), 86.3% vs. 87.2% (*p* = 0.50) respectively). However, the AIC/LASSO model outperformed the optimised expert model in the retrospective dataset (AUC of 87.2 vs. 84.1, *p* = 0.03) (Fig. 3). qXR feature-level performance against radiologist annotation is provided in supplementary materials (S8 and S9).

**Table 4:** Performance statistics for each model evaluated over the training, test and retrospective datasets at the Youden index threshold and 15% flag rate

	Baseline expert			Optimised expert (five features)						AIC/ LASSO feature selection (four features)					
Dataset	Training	Test	Retro	Training		Test		Retro		Training		Test		Retro	
AUC (%) (95% CI)	70.2 (66.0, 74.3)	67.5 (61.8, 73.2)	68.1 (63.0, 73.3)	83.4 (80.1, 86.7%)		86.3 (82.2, 90.4)		84.1 (80.0, 88.2)		84.8 (81.5, 88.1)		87.2 (82.7, 91.7)		87.2 (84.0, 90.5)	
**Threshold**	**Each feature at vendor recommended threshold**			**Youden**	**15% flag rate**^**a**^	**Youden**	**15% flag rate**^**a**^	**Youden**	**15% flag rate**^**a**^	**Youden**	**15% flag rate**^**a**^	**Youden**	**15% flag rate**^**a**^	**Youden**	**15% flag rate**^**a**^
TP	75	33	47	109	87	55	43	72	63	121	94	64	46	88	77
TN	1573	826	774	1436	1552	759	819	645	728	1371	1559	733	826	566	710
FP	178	76	113	315	199	143	83	242	159	380	192	169	76	321	177
FN	76	44	49	42	64	22	34	24	33	30	57	13	31	8	19
Sens (%) (95% CI)	49.7 (41.4, 57.9)	42.9 (31.6, 54.6)	49.0 (38.6, 59.4)	72.2 (64.3, 79.2)	57.6 (49.3, 65.6)	71.4 (60.0, 81.2)	55.8 (44.1, 67.2)	75.0 (65.1, 85.3)	65.6 (55.2, 75.0)	80.1 (72.9, 86.2)	62.3 (54.0, 70.0)	83.1 (72.9, 90.7)	59.7 (47.9, 70.8)	91.7 (84.2, 96.3)	80.2 (70.8, 87.6)
Spec (%) (95% CI)	89.8 (88.3, 91.2)	91.6 (89.6, 93.3)	87.3 (84.9, 89.4)	82.0 (80.1, 83.8)	88.6 (87.1, 90.1)	84.1 (81.6, 86.5)	90.8 (88.7, 92.6)	72.7 (69.7, 75.6)	82.1 (79.4, 84.5)	78.3 (76.3, 80.2)	89.0 (87.5, 90.5)	81.3 (78.6, 83.8)	91.6 (89.6, 93.3)	63.8 (60.6, 67.0)	80.0 (77.3, 82.6)
PPV (%) (95% CI)	29.6 (24.1, 35.7)	30.3 (21.8, 39.8)	29.4 (22.4, 37.1)	25.7 (21.6, 30.1)	30.4 (25.0, 36.1)	27.8 (21.7, 34.6)	34.1 (25.9, 43.1)	22.9 (18.4, 28.0)	28.4 (22.5, 34.8)	24.2 (20.5, 28.1)	32.9 (27.5, 38.6)	27.5 (21.8, 33.7)	37.7 (29.1 46.9)	21.5 (17.6, 25.8)	30.3 (24.7, 36.4)
NPV (%) (95% CI)	95.4 (94.3, 96.4)	94.9 (93.3, 96.3)	94.0 (92.2, 95.6)	97.2 (96.2, 97.9)	96.0 (95.0, 96.9)	97.2 (95.8, 98.2)	96.0 (94.5, 97.2)	96.4 (94.7, 97.7)	95.7 (94.0, 97.0)	97.9 (97.0, 98.6)	96.5 (95.5, 97.3)	98.3 (97.0, 99.1)	96.4 (94.9 97.5)	98.6 (97.3, 99.4)	97.4 (96.0, 98.4)
Accuracy (%) (95% CI)	86.6 (85.0, 88.1)	87.7 (85.0, 88.1)	83.5 (81.1, 85.8)	81.2 (79.4, 83.0)	86.2 (84.5, 87.7)	83.1 (80.7, 85.4)	88.0 (85.9, 90.0)	72.9 (70.0, 75.7)	80.5 (77.8, 82.9)	78.4 (76.5, 80.3)	86.9 (85.3, 88.4)	81.4 (78.8, 83.8)	89.1 (86.9 91.0)	66.5 (63.5, 69.5)	80.1 (77.4, 82.5)
Flag rate at youden index (%)	13.3% (11.8% 14.9%)	11.1% (9.2%, 13.3%	16.3 (14.0, 18.7)	22.3 (20.4, 24.2)	15.0 (13.5, 16.7)	20.2 (17.8, 22.9)	12.9 (10.8, 15.5)	31.9 (29.0, 35.0)	22.6 (20.0, 25.3)	26.3 (24.4, 28.4)	15.0 (13.5, 16.7)	23.8 (21.2, 26.6)	12.5 (10.5 14.7)	41.6 (38.5, 44.8)	25.8 (23.1, 28.7)

^a^ Threshold for 15% flag rate is calculated on the training set and then applied to the test and retrospective sets

*AUC* area under the curve, *CI* confidence interval, *TP* true positive, *TN* true negative, *FP* false positive, *FN* false negative, *PPV* positive predictive value, *NPV* negative predictive value

### Model comparison: Youden index (best overall sensitivity/specificity performance) threshold

**Fig. 4:**
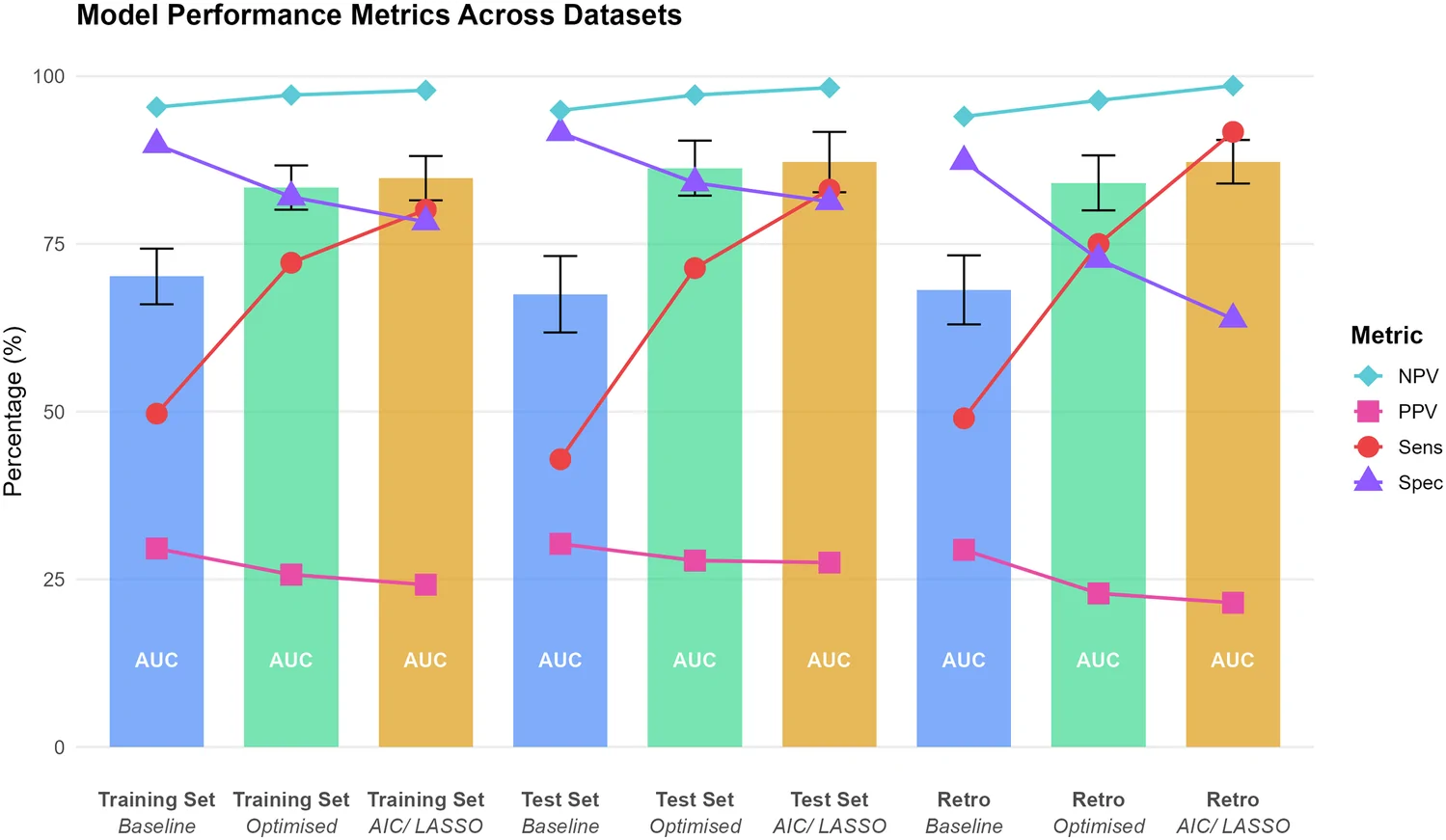
Model comparison between training, test and retrospective validation sets. AUC is represented by the height of the bar

The AIC/LASSO model was the highest performing for sensitivity, achieving statistically significant superiority over the optimised expert model across all datasets using McNemar’s test (80.1% vs. 72.2% (*p* = 0.027) in the training set, 83.1% vs. 71.4% (*p* = 0.027) in the test set, 91.7% vs. 75.0% (*p* < 0.01) in the retrospective set). The optimised expert was the highest performing for specificity, achieving statistically significant superiority over the AIC/LASSO model across all datasets (82.0% vs. 78.3% (*p* < 0.01) in the training set, 84.2% vs. 81.3% (*p* < 0.01) in the test set, 72.3% vs. 63.8% (*p* < 0.01) in the retrospective set. Model comparison is illustrated in Fig. 4.

### Model comparison: 15% flag rate threshold

There were no statistically significant differences in sensitivity between the optimised expert and AIC/LASSO model in the training and test set using McNemar’s test (57.6% vs. 62.2% (*p* = 0.16) in the training set, 55.8% vs. 59.7% (*p* = 0.47) in the test set). The AIC/ LASSO model outperformed the optimised expert model in the retrospective dataset (80.2% vs. 65.6% (*p* = 0.04)).

There were no statistically significant differences in specificity between the optimised expert and AIC/LASSO model over all datasets (88.6% vs. 89.0% (*p* = 0.48) in the training set, 90.8% vs. 91.6% (*p* = 0.29) in the test set, 82.1% vs. 80.1% (*p* = 0.08) in the retrospective dataset).

## Discussion

We evaluated the impact of feature selection and thresholding on a commercial CXR algorithm and its performance in detecting USC. Through regression modelling, we identified high-performing features and proposed composite models achieving significant improvement over an expert-selected feature list with vendor-derived thresholds calibrated on our local population. CXR algorithm detection of thoracic malignancy has a wide range of reported performance, depending on the research question, reference standard and population [22]; there is no established benchmark for USC detection. Our work underscores the importance of local capability in validating and refining algorithms for the target population and endpoint.

There are significant flaws in extrapolating feature nomenclature from a clinical setting to a commercially derived algorithm label, as exemplified in this work by the weak performance of malignancy-associated qXR features such as mass, hilar enlargement and mediastinal widening in our regression models. Conversely, we saw an unexpectedly strong performance of the Tuberculosis feature.

These findings highlight the nature of multi-classification algorithms for researchers, wherein each feature is an independently developed model that predicts the presence or absence of an annotation. These algorithms are currently unable to exercise clinical reasoning or incorporate local disease prevalence and are prone to collinearity in their prediction scores, i.e., simultaneously scoring a lesion as mass, cavity, tuberculosis and nodule. Whilst these algorithms have enormous classification capability within their networks, the final feature definitions reflect developer-generated label structures rather than population patterns. In our study, for example, Tuberculosis is a strong predictive feature not because of any biological association with USC or its true presence in our datasets, but because there are overlapping patterns between images that radiologists label as USC and the way Tuberculosis was labelled in the algorithm training set. This effect is likely compounded where a feature has been developed in a population with a different disease prevalence to the deployment setting, representing an inherent difficulty in transferring algorithm features between patient populations. Caution is therefore warranted when extrapolating feature-performance metrics to new clinical settings or tasks.

We explored translational considerations in thresholding and flagging rate. Firstly, combined feature models with a single threshold had significantly higher performance than individually thresholded features (Fig. 4). This method offers intuitive calibration for real-world deployment and post-market surveillance in clinical pathways while preserving data-driven feature weighting. Secondly, we explore the impact of thresholding models to limit positive flagging at the expense of optimal classification performance. Alert fatigue may undermine clinical engagement by saturating radiologist capacity and reducing clinician engagement [23, 24]. Our 15% threshold resulted in a mean reduction of 15.4% in sensitivity and a mean increase of 10.0% in specificity across all datasets for both the optimised expert model and the AIC/ LASSO model. Decision-makers must ‘trade off’ performance against clinical workload when setting thresholds for diagnostic technology and conduct independent analyses for their specific use cases.

### Limitations and future work

Feature outputs are specific to individual models; this work presents an exemplar methodology for optimal calibration of a specific product in a single-centre population. Whilst the methodology is generalisable, the reported results are not.

Our image annotations for USC are derived from a dedicated research platform and therefore cannot be considered truly “real-world”. On this platform, radiologists lacked access to clinical details or prior imaging. Therefore, chronic abnormalities and known malignancies would be flagged as USC rather than being correctly identified as false positives. Radiologists were also restricted to a defined feature set rather than the typical free-text report. Whilst this allows for a focus on ‘pixel-level’ abnormalities, it does not represent real-world and highlights a key limitation of current CXR AI technology.

Future work would benefit from the inclusion of a real-world clinical USC label as an additional layer for comparison, along with further refinement of the feature optimisation methodology and the development of guidelines for pre-deployment calibration on a local population. More broadly, AI CXR performance may be optimised for this use case by training a dedicated primary USC feature against radiologist annotations rather than using combinations of existing features. The increasing use of CXR foundation models may make this possible in both vendor and academic environments.

## Conclusion

This work explores optimal feature selection for detecting USC in a West of Scotland population using a commercial CXR algorithm. We highlight the importance of post-market calibration to augment algorithm capability and demonstrate the potential to develop specific regression-based wrapper models for use in a local clinical setting. We address translational challenges in post-market surveillance and the impact of thresholding on clinical capacity on performance.

## Supplementary information

ELECTRONIC SUPPLEMENTARY MATERIAL

## Data Availability

The reported datasets are available through the West of Scotland Safe Haven.

## Abbreviations

AI: Artificial/intelligence
AIC: Akaike Information Criterion
AUC: Area under curve
CXR: Chest X-ray
LASSO: Least absolute shrinkage and selection operator
NHSGGC: NHS Greater Glasgow and Clyde
RADICAL: Radiograph accelerated detection and identification of cancer in the lung
USC: Urgent suspicion of cancer
