# Supplementary material for: Flagging urgent suspicion of lung cancer with AI CXR algorithms: optimising feature selection and performance
Source: Insights Imaging. 2026 Oct 31;17:272. doi: 10.1186/s13244-026-02438-7 (PMC13723462; doi:10.1186/s13244-026-02438-7)
Supplement: Supplementary file 1 [file 13244_2026_2438_MOESM1_ESM.pdf]

ELECTRONIC SUPPLEMENTARY MATERIAL

Flagging Urgent Suspicion of Lung Cancer with AI CXR Algorithms: Optimising Feature Selection and Performance

Insights Imaging (2026) Duncan SF, Dudman K, Odigbo S et al.

S1- About qXR

qXRv4.0, serving as a workflow aid, integrates into the Radiologist's reporting process. It identifies abnormalities within the lungs by pinpointing the specific regions of concern and prioritising chest X-rays for review. These findings are presented as Secondary Captures (Figure 1S), comprising single series with outlined and labelled target abnormalities. These captures can be viewed independently on Radiology PACS as thumbnails and assign a Triage priority on CRIS. A dataset containing 4.2 million Chest X-rays was used for training and validating the algorithms, it is based on deep learning algorithms, a form of machine learning where the hypothesis set is composed of neural networks (Convolutional Neural Networks in this case). qXRv4.0 consists of separate detection pipelines for each target abnormality, as there are several details pertaining to the location, size and texture that vary across abnormalities. qXR has undergone training to identify artefacts and suboptimal X-rays.

Commented [GU1]: American English form used here but British English later ("artefacts" 8 lines below). I think Eur Rad allows either but has to be consistent.

qXR will only process frontal (PA/AP view) CXR images, which have a minimum 1440×1440 resolution with a complete chest view. The performance of qXR will be affected by images taken with inadequate exposure and inspiration, images that are inverted/flipped/rotated, images with an incomplete view of chest or lung parenchyma and images with significant artefacts.

If qXR rejects a CXR image due to sub-optimal image quality issues, then rejection reasons will be logged for further analysis. Malfunctions in the qXR device could occur through incorrect pre-processing of the image data or incorrect identification of features. These may be logged in the device error log, which will be investigated by Qure.ai.

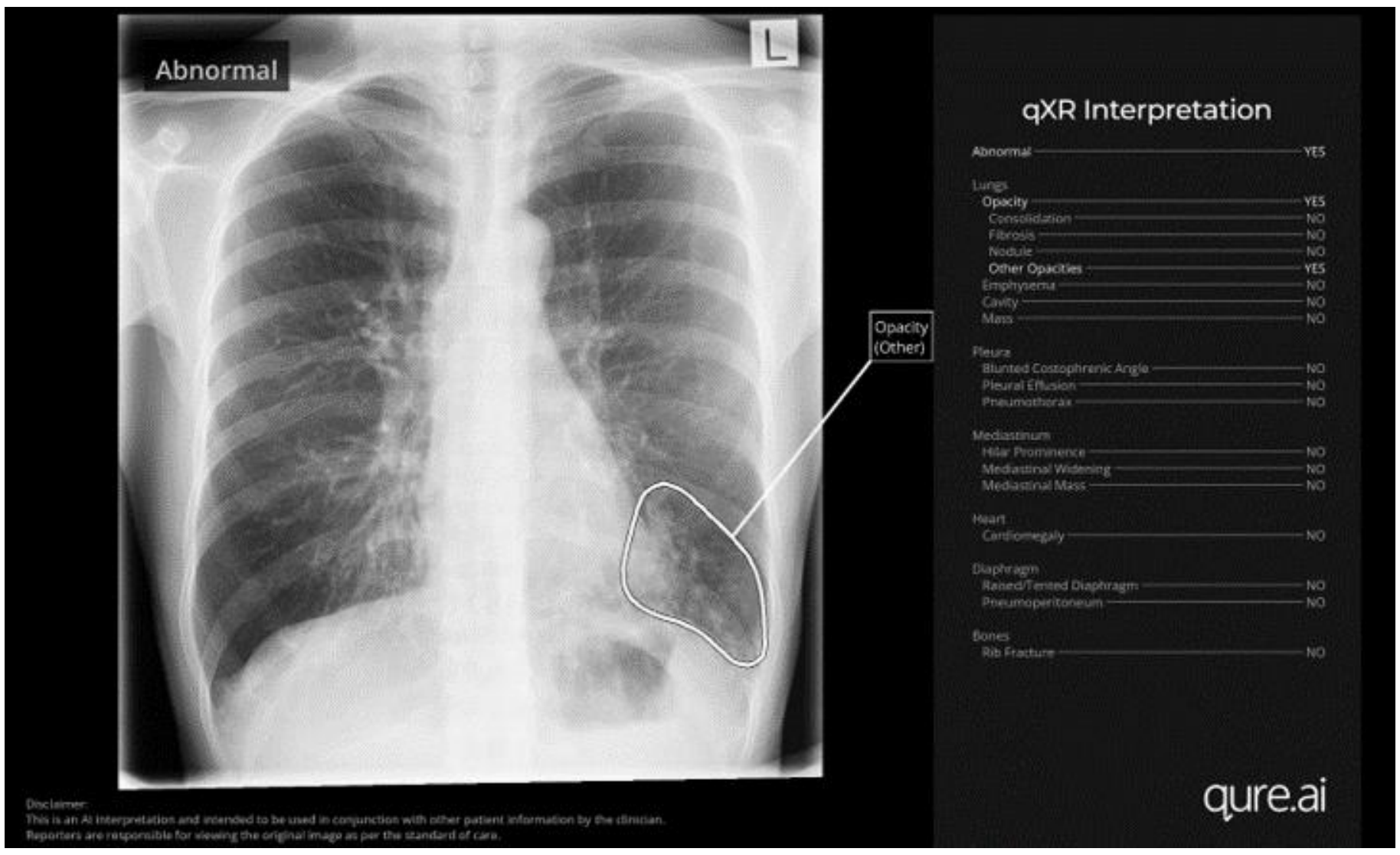

Figure 1S- qXR sample image with overlay

S2- About qStudy

qStudy is Qure.ai's DICOM annotation platform.

The annotating radiologists viewed each case, with software similar to a clinical image reporting system. The annotator assessed for the presence of any abnormalities on the radiograph and, if identified, select all the relevant from a list. (Figure 2S).

Commented [GU2]: Need to be clearer they selected the most appropriate descriptor from a list. Could be read as they drew a region of interest on the image, especially as the image above shows an ROI annotation.

Radiologists were specifically asked "Do the CXR findings show signs suspicious for cancer? (USC)" with the following definition provided for clarification:

"This is used to prioritise the case for urgent reporting. If you think the chest radiograph shows signs suspicious for cancer, please choose "Yes" from the drop-down menu, otherwise select "No"."

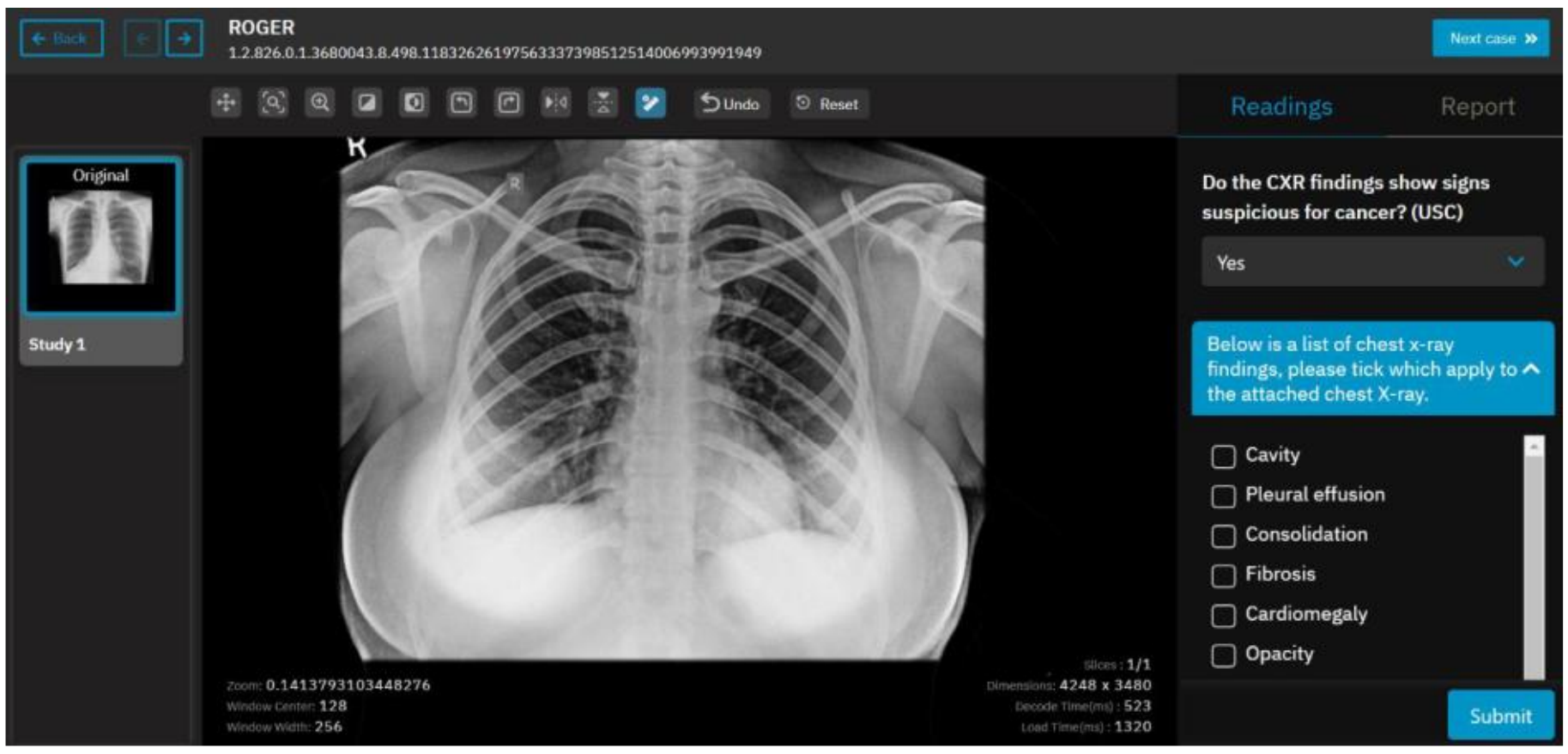

Figure 2S- qStudy Screen capture

S3- Qure.ai calibration exercise methodology for threshold determination

The clinical team selected 500 CXRs enriched to a 50% USC prevalence, identified using the presence or absence of a cancer-tracking code assigned during usual-care reporting. These scans were processed through qXR for AI-generated probability scores and uploaded to qStudy for reference-standard annotation by NHS GGC radiologists. The combined dataset of ground-truth labels and AI outputs was then reviewed internally by Qure.ai.

For each feature proposed for USC classification, qXR's continuous probability scores were compared with the binary ground-truth labels to assess diagnostic behaviour. Sensitivity, specificity, PPV, and NPV were evaluated across a range of potential probability cut-offs. By iteratively varying the threshold and reviewing the resulting FN and FP patterns, Qure.ai identified pathology-specific operating points that provided an appropriate balance for the NHS GGC population, with emphasis on minimising false negatives in the lung cancer context. These thresholds were subsequently adopted for use in the main study. A comparison of study specific vs default thresholds is provided below for the 5 features used in the baseline expert model.

USC feature	Initial/default threshold	Baseline expert threshold following qure calibration
Cavity	0.90	0.70
Mediastinal widening	0.50	0.15
Nodule	0.65	0.70
Mass	0.50	1.00
Hilar enlargement	0.75	0.70

S4- Features excluded from AIC/ LASSO regression with rationale

- **Reticulonodular pattern**- Limited clinical relevance for new diagnosis of lung cancer
- **Opacity**- An ‘umbrella feature’ which is comprised of a combination of other feature scores rather than a dedicated individual annotation process.
- **Pneumothorax-** Low incidence finding which may be a confounder for USC detection (due to association with biopsy).
- **Pneumoperitoneum-** Low incidence finding with no clinical relevance to USC detection.
- **Abnormal**- Non-specific meta-feature for alternative use case.

S5- Interobserver disagreements rates in the prospective dataset

Feature	Disagreements (n)	Disagreement rate (%)
Normal*	1038	36.0
Nodule	840	29.2
Atelectasis	617	21.5
USC	343	11.9
Opacity	327	11.4
Blunted costophrenic angle	316	11.0
Consolidation	205	7.1
Cardiomegaly	193	6.7
Hyperinflation	179	6.2
Hilar enlargement	139	4.8
Pleural effusion	122	4.2
Mass	108	3.8
Elevated hemidiaphragm	86	3.0
Reticulonodular pattern	68	2.4
Mediastinal widening	62	2.2
Fibrosis	59	2.1
Rib fracture	48	1.7
Cavity	21	0.7
Tuberculosis	11	0.4
Pneumothorax	4	0.1
Pneumoperitoneum	3	0.1

Interobserver disagreement rates for ground-truthed features in prospective dataset. Values represent the number and proportion of cases where the two independent readers disagreed. 3rd reader adjudication was only conducted for disagreements in USC status.

*Normal was not a feature output provided by qXR.

S6- Threshold-based USC detection performance of individual baseline expert features.

Training set performance is reported for each individual feature selected in the baseline expert model. Thresholds were defined by the vendor following an internal calibration exercise (S3). Baseline expert model is reported (as seen in main article Table 4) alongside a 4-feature model with mediastinal widening excluded. No mass predictions reached threshold in retrospective data.

Feature / Model	Threshold	Training cohort				Test cohort				Retrospective cohort			
		Sens % (95%CI)	Spec % (95%CI)	PPV % (95%CI)	NPV % (95%CI)	Sens % (95%CI)	Spec % (95%CI)	PPV % (95%CI)	NPV % (95%CI)	Sens % (95%CI)	Spec % (95%CI)	PPV % (95%CI)	NPV % (95%CI)
Cavity	**0.70**	**11.9** (7.2, 18.2)	**99.4** (99.0, 99.7)	**64.3** (44.1, 81.4)	**92.9** (91.6, 94.0)	**9.1** (3.7, 17.8)	**99.4** (98.7, 99.8)	**58.3** (27.7, 84.8)	**92.8** (90.9, 93.9)	**7.3** (3.0, 14.4)	**99.1** (98.2, 99.6)	**46.7** (21.3, 73.4)	**90.8** (88.8, 92.6)
Nodule	**0.70**	**37.7** (30.0, 46.0)	**93.4** (92.1, 94.5)	**32.9** (26.0, 40.5)	**94.6** (93.4, 95.6)	**31.2** (21.1, 42.7)	**95.7** (94.1, 96.9)	**38.1** (26.1, 51.2)	**94.2** (92.5, 95.6)	**38.5** (28.8, 49.0)	**94.0** (92.3, 95.5)	**41.1** (30.8, 52.0)	**93.4** (91.6, 94.9)
Mass	**1.00**	**2.6** (0.7, 6.6)	**99.8** (99.5, 100.0)	**57.4** (18.4, 90.1)	**92.2** (90.9, 93.4)	**1.3** (0.0, 7.0)	**99.7** (99.0, 99.9)	**25.0** (0.6, 80.6)	**92.2** (90.3, 93.8)	N/A	N/A	N/A	N/A
Hilar enlargement	**0.70**	**7.9** (4.2, 13.5)	**97.4** (96.5, 98.1)	**20.7** (11.2, 33.4)	**92.5** (91.2, 93.6)	**6.5** (2.1, 14.5)	**97.3** (96.1, 98.3)	**17.2** (5.8, 35.8)	**92.4** (90.6, 94.0)	**4.2** (1.1, 10.3)	**95.8** (94.3, 97.0)	**9.8** (2.7, 23.1)	**90.2** (88.2, 92.1)
Mediastinal widening	**0.15**	**0.7** (0.0, 3.6)	**99.3** (98.8, 99.6)	**7.7** (0.2, 36.0)	**92.1** (90.7, 92.7)	**5.2** (1.4, 12.8)	**98.9** (98.0, 99.5)	**28.6** (8.4, 58.1)	**92.4** (90.6, 94.0)	**4.2** (1.1, 10.3)	**97.0** (95.6, 98.0)	**12.9** (3.6, 29.8)	**90.3** (88.3, 92.1)
Baseline expert model (5 features)	**≥1 of 5**	**49.7** (41.4, 57.9)	**89.8** (88.3, 91.2)	**29.6** (24.1, 35.7)	**95.4** (94.3, 96.4)	**42.9** (31.6, 54.6)	**91.6** (89.6, 93.3)	**30.3** (21.8, 39.8)	**94.9** (93.3, 96.3)	**49.0** (38.6, 59.4)	**87.3** (84.9, 89.4)	**29.4** (22.4, 37.1)	**94.0** (92.2, 95.6)
4 feature model with mediastinal widening excluded	**≥1 of 4**	**49.0** (40.8, 57.3)	**90.5** (89.1, 91.9)	**30.8** (25.1, 37.1)	**95.4** (94.2, 96.3)	**41.6** (30.4, 53.4)	**92.4** (90.4, 94.0)	**31.7** (22.8, 41.7)	**94.9** (93.2, 96.2)	**44.8** (34.6, 55.3)	**89.6** (87.4, 91.6)	**31.9** (24.1, 40.4)	**93.8** (91.9, 95.3)

S7- AUC comparison for AIC/LASSO model.

Pairwise AUC comparison (table 1S) of the AIC feature model, LASSO lambda(min) model and LASSO lambda(1se) using the DeLong's test with Bonferroni correction did not reveal any significant differences in performance between the three candidate models. In accordance with our pre-specified rule, the model with the fewest features (four-feature LASSO lambda(1se)) was therefore selected as the AIC/LASSO model for final comparison.

Feature selection model	Included features	AUC (95% CI)	
		Training	Test
AIC	Cavity<br>Nodule<br>TB<br>Consolidation<br>Hilar enlargement	**85.4** (82.2, 88.5)	**88.0** (83.9, 92.0)
LASSO (lambda= min)	Cavity<br>Mass<br>Nodule<br>TB<br>Consolidation<br>Hilar enlargement	**85.3** (82.2-88.4)	**87.9** (83.8, 92.0)
LASSO (lambda= 1se)	Cavity<br>Nodule<br>TB<br>Consolidation	**84.8** (81.5, 88.1)	**87.2** (82.7, 91.7)

Feature selection model comparison	Unadjusted p-value	Bonferroni-adjusted p-value
AIC vs LASSO (lambda=min)	0.39	1.00
AIC vs LASSO (lambda=1se)	0.24	0.71
LASSO (lambda=min) vs LASSO (lambda= 1se)	0.30	0.90

S8- qXR feature level AUC against USC radiologist annotation

qXR Feature	qXR score vs USC (AUC)	AUC 95% CI
Cavity	0.79	0.76-0.83
Nodule	0.78	0.75-0.82
Mediastinal Widening	0.44	0.41-0.48
Hilar enlargement	0.56	0.51-0.60
Opacity	0.72	0.68-0.77
Fibrosis	0.72	0.69-0.76
Blunted costophrenic angles	0.65	0.61-0.69
Elevated hemidiaphragm	0.61	0.58-0.65
Hyperinflation	0.66	0.62-0.70
Pleural effusion	0.66	0.62-0.71
Atelectasis	0.67	0.63-0.71
Rib fracture	0.58	0.54-0.62
Pneumothorax	0.63	0.59-0.66
TB	0.83	0.80-0.86
Consolidation	0.76	0.73-0.80
Pneumoperitoneum	0.42	0.38-0.46
Reticulonodular pattern	0.73	0.69-0.76
Mass	0.59	0.55-0.63
Cardiomegaly	0.54	0.50-0.58
Calcification	0.73	0.70-0.77
Mediastinal Mass	0.43	0.39-0.47

Analysis conducted over entire prospective data set.

S9- qXR feature level performance against ground truth reader annotations

qXR Feature	qXR score vs reader 1 (AUC)	qXR score vs reader 2 (AUC)	qXr score vs reader 3 (AUC)*
Cavity	0.86	0.77	0.66
Nodule	0.65	0.60	0.73
Mediastinal Widening	0.77	0.72	0.72
Hilar enlargement	0.60	0.59	0.53
Opacity	0.82	0.73	0.61
Fibrosis	0.85	0.89	0.83
Blunted costophrenic angles	0.66	0.68	0.47
Elevated hemidiaphragm	0.94	0.92	0.91
Hyperinflation	0.90	0.89	0.90
Pleural effusion	0.89	0.91	0.98
Atelectasis	0.58	0.59	0.66
Rib fracture	0.94	0.83	0.95
Pneumothorax	0.74	0.99	NA
TB	0.98	0.95	0.81
Consolidation	0.88	0.86	0.85
Pneumoperitoneum	0.98	0.73	0.99
Reticulonodular pattern	0.91	0.91	0.95
Mass	0.59	0.60	NA
Cardiomegaly	0.90	0.90	0.93

Reader 3 only used when disagreement with 1 and 2.

“Calcification”, “Mediastinal mass” and “Abnormal” feature not scored or ground truthed.
